# A new holistic 3D non-invasive analysis of cellular distribution and motility on fibroin-alginate microcarriers using light sheet fluorescent microscopy

**DOI:** 10.1371/journal.pone.0183336

**Published:** 2017-08-17

**Authors:** Serena Duchi, Filippo Piccinini, Michela Pierini, Alessandro Bevilacqua, Maria Luisa Torre, Enrico Lucarelli, Spartaco Santi

**Affiliations:** 1 Osteoarticolar Regeneration Laboratory, Rizzoli Orthopaedic Institute, Bologna, Italy; 2 Department of Surgery, St Vincent’s Hospital, University of Melbourne, Fitzroy, Victoria, Australia; 3 Istituto Scientifico Romagnolo per lo Studio e la Cura dei Tumori (IRST) S.r.l., IRCCS, Meldola (FC), Italy; 4 Department of Biomedical and Neuromotor Sciences (DIBINEM), Alma Mater Studiorum University of Bologna, Bologna, Italy; 5 Advanced Research Center on Electronic Systems “Ercole De Castro” (ARCES), Alma Mater Studiorum University of Bologna, Bologna, Italy; 6 Department of Computer Science and Engineering (DISI), Alma Mater Studiorum University of Bologna, Bologna, Italy; 7 Cell Delivery System Lab, Department of Drug Sciences, University of Pavia, Pavia, Italy; 8 Institute of Molecular Genetics (CNR), Bologna, Italy; 9 SC Laboratory of Musculoskeletal Cell Biology, Rizzoli Orthopaedic Institute, Bologna, Italy; Chang Gung University, TAIWAN

## Abstract

Cell interaction with biomaterials is one of the keystones to developing medical devices for tissue engineering applications. Biomaterials are the scaffolds that give three-dimensional support to the cells, and are vectors that deliver the cells to the injured tissue requiring repair. Features of biomaterials can influence the behaviour of the cells and consequently the efficacy of the tissue-engineered product. The adhesion, distribution and motility of the seeded cells onto the scaffold represent key aspects, and must be evaluated *in vitro* during the product development, especially when the efficacy of a specific tissue-engineered product depends on viable and functional cell loading. In this work, we propose a non-invasive and non-destructive imaging analysis for investigating motility, viability and distribution of Mesenchymal Stem Cells (MSCs) on silk fibroin-based alginate microcarriers, to test the adhesion capacity of the fibroin coating onto alginate which is known to be unsuitable for cell adhesion. However, in depth characterization of the biomaterial is beyond the scope of this paper. Scaffold-loaded MSCs were stained with Calcein-AM and Ethidium homodimer-1 to detect live and dead cells, respectively, and counterstained with Hoechst to label cell nuclei. Time-lapse Light Sheet Fluorescent Microscopy (LSFM) was then used to produce three-dimensional images of the entire cells-loaded fibroin/alginate microcarriers. In order to quantitatively track the cell motility over time, we also developed an open source user friendly software tool called *Fluorescent Cell Tracker in Three-Dimensions* (*F-Tracker3D*). Combining LSFM with *F-Tracker3D* we were able for the first time to assess the distribution and motility of stem cells in a non-invasive, non-destructive, quantitative, and three-dimensional analysis of the entire surface of the cell-loaded scaffold. We therefore propose this imaging technique as an innovative holistic tool for monitoring cell-biomaterial interactions, and as a tool for the design, fabrication and functionalization of a scaffold as a medical device.

## Introduction

The purpose of tissue engineering and regenerative medicine is to replace tissue lost or damaged as a consequence of cancer, diseases, trauma, congenital abnormalities, and other pathologies [1]. Biomaterials serve as scaffolds to deliver cells and provide both biological signals and physical support for the cells [2]. The synergism that exists between stem cell biology and biomaterials aims to generate a profound improvement to stem cell-based clinical applications used in tissue engineering. However, the number of stem cell biomaterial-based clinical trials are still limited and the outcomes are not optimized [3]. There are distinct challenges in all facets of this research, given the unique environment resulting from the presence of biomaterials and cells, such as the methods of monitoring and assessing the produced engineered constructs.

Sophisticated chemistries are used to synthesize materials that mimic and modulate native tissue microenvironments, and at the same time are able to structurally support the cells’ adhesion and distribution on the biomaterial [4]. Ideally, a uniform distribution of the cells on the surface or within the scaffold would enable the cells to reach the maximal load on the construct and also to obtain an efficient delivery of cells to the injured site.

An example of a biomaterial that requires complex chemistries to improve cell adhesion capacity is alginate. Alginate microcarriers are noteworthy targets for applications in tissue regenerative medicine due to their good biocompatibility and mechanical properties [5]. In the recent years, alginate microcarriers have also been used as an injectable biomaterial to directly deliver a variety of *in vivo* cells (keratinocytes, fibroblast, and mesenchymal stem/stromal cells), as a resorbable vehicle for biomolecules and drugs for therapeutics and tissues repair [6].

However, the surface of alginate is unsuitable for cell adhesion due to the presence of negative charges and the deficiency of integrin domains [7][8], thus preventing the ability of cells to proliferate and acquire their proper morphology. This therefore leads to dysfunctional behavior and function. These findings suggest that the addition of bioactive molecules to alginate will improve their ability to act as cell carriers [9]. In this regard, silk-fibroin, a fibrous polymer derived from different silkworm species, has been widely used as suitable matrix/substrate due to its high biocompatibility, excellent mechanical properties and abundance of cell binding motifs (arginine-glycine-aspartic acid, RGD) in its structure, which enhance cell attachment and proliferation [10][11][12]. However, only a few papers demonstrated the improved adhesion of the cells onto silk-fibroin coated alginate microcarriers.

Currently, the methods used to characterize the surface of the scaffold are invasive, cause destruction of the sample and don’t allow for an accurate assessment of the entire surface of the construct. At present, the morphology of the adherent cells on the surfaces of tissue engineered constructs can be typically observed using scanning electron microscopy (SEM) [13][14] and confocal fluorescent microscopy after cell labelling [15]. Transmission electron microscopy (TEM) has been used to observe detailed cellular—scaffold information [16]. However, it would be difficult to translate such high-resolution images into consistent cell-distribution results. Considering the light reflection, opacity, dimensions, sample preparation, microscopic visualization of viable and dead cells, and distribution of cells without disruption of the scaffold, the current methods of visualization of the entire structure of the sample are limited to a portion of the scaffold or to the three-dimensional reconstruction from single images. In addition, the visual depth of the confocal laser-scanning microscope is often limited to less than 500 μm depending on the material. Alternative approaches to quantify cell distribution in tissue engineered scaffolds include micro-computed tomography and magnetic resonance imaging. However, they require the use of magnetic iron oxide nanoparticles entrapped in seeded cells [17]. Therefore, imaging techniques for three-dimensional (3D) analyses have been identified as a strategic priority in tissue engineering scaffolds, in order to enable non-destructive, quantitative, and 3D observations [18].

Light Sheet Fluorescence Microscopy (LSFM) has been proposed as an alternative approach to the traditional techniques since it displays the above mentioned features for imaging analyses [19]. LSFM is a fluorescence microscopy technique in which the illumination laser beam is shaped into a thin plane of light (usually a few hundred nanometers to a few micrometers), and the illumination of the sample takes place perpendicularly to the detection objective. Compared to confocal microscopy, this method minimizes the photodamage and stress induced on a living sample, reduces the background signal and thus creates images with higher contrast. In addition, with LSFM it is possible to obtain multiple views along different angles that can be combined together so that hidden parts of the sample become visible. A detailed 3D volume reconstruction of the sample is thus achievable, even though this feature is not typically offered in conventional multidimensional microscopy imaging systems.

LSFM is also well suited for imaging large live specimens over long periods of time and is currently established as the leading tool for the study of the development of organisms *in toto*: zebrafish, fly and mouse embryo [20][21] at subcellular resolution. However in larger animals, tissues or organs [22][23], 3D cell cultures and spheroids [24][25] transparency and laser light scatter/absorption becomes a major obstacle in achieving refractive uniformity throughout the specimen. Therefore fixation and chemical clearing are required to allow greater depth of imaging. However, LSFM imaging used to verify the distribution of stem cells seeded onto scaffold biomaterials is still an unexplored field of application. Accordingly, given that LSFM imaging strategies offer high speeds, large and deep fields of view and long-term imaging capacity, we decided to characterize Mesenchymal Stem/Stromal Cells (MSCs) behaviour onto fibroin-coated alginate microcarriers to evaluate the hypothesised adhesion capacity of silk-fibroin to allow MSCs loading onto alginate scaffolds surface. Through LIVE/DEAD^®^ labelling onto the whole scaffold and LSFM image acquisition, we evaluated cell adhesion, distribution and motility during time in culture.

A 3D tracking system was needed to monitor the cell motility on the MSCs-loaded microcarriers. Today, few systems are available for tracking cells/particles in 3D starting from a time-lapse dataset of *z*-stacks of images [26]. The *ImarisTrack* module (http://www.bitplane.com/imaris/imaristrack) of *IMARIS* (Bitplabe, Zurich, Switzerland) is the most complete solution in the field and is used for a wide range of applications, ranging from bacterial to cancer cell analysis [27]. This software suite provides the user with several low-level routines, permitting the development of individualised custom, high-level, procedures. However, besides being commercially available, it requires good image processing skills to understand how the single routines have to be connected so as to achieve the expected behaviour. On the other hand, *ImageJ* (https://imagej.net/Welcome) is a freely available image processing tool, with several plugins for processing *z*-stack of images and tracking cells/particles in 3D. Again, it does not include a ready-to-use procedure and basic programming skills are required to concatenate more plugins suitably and process the input dataset. In order to equip biologists with an off-the-shelf and user-friendly tool to effectively monitor the cell motility on the MSCs-loaded microcarriers, we developed *Fluorescent Cell Tracker in Three-Dimensions* (*F-Tracker3D*). It is an open-source software providing automatic, semi-automatic and manual methods to track single cells in time-lapse LSFM acquisitions (https://sourceforge.net/p/f-tracker3d). *F-Tracker3D* extends to 3D the automatic, semi-automatic and manual methods available in *CellTracker* [28] (www.celltracker.website), an open-source software tool recently developed for tracking MSCs, cancer cells, and many other types of cells in 2D [29][30][31].The tutorial together with the on-line user manual makes it easy to use.

The combination of LSFM and *F-Tracker3D* allowed us to develop a holistic analysis of MSCs loaded onto silk-fibroin coated alginate microcarriers by defining and measuring, at a cellular level, the direction and the path length covered by the cells during several hours of observation, without damaging the sample.

## Materials and methods

### Alginate silk-fibroin coated microcarriers

Alginate microcarriers were produced according to the following procedure: briefly, a 1% w/v sodium alginate (Sigma-Aldrich, Milan, Italy) aqueous solution was dropped into a bath of 100 mM calcium chloride (Sigma-Aldrich) water solution using an automatic encapsulator (Encapsulator VAR V1, Nisco Engineering AG, Zurich, Switzerland, 0.17-mm diameter nozzle). *Bombyx mori* cocoons were degummed in autoclave, dried at room temperature, and solubilized in phosphoric acid/formic acid (80:20 v/v) (Sigma-Aldrich). Fibroin solution was dialyzed against water (polyethersulfone membrane, cut off 12 kDa, Visking, London, UK), obtaining a 1.5% w/v silk fibroin aqueous solution. Alginate microcarriers coated with silk fibroin were obtained adding alginate microcarriers into silk fibroin solution for 5 min. Microcarriers were collected by filtration and immersed in 96% (v/v) ethanol (Carlo Erba Reagents, Milan, Italy) for 15 min to induce silk conformational transition. Silk/alginate microcarriers presented β-sheet fibroin conformation (verified by Fourier Transform Infrared Spectroscopy), spherical geometry, and average diameter of about 400 μm in laser light scattering analyzer (Mastersizer 2000, Malvern Instruments Ldt, Worcestershire, UK).

### Mesenchymal Stem/Stromal Cells (MSCs) isolation, characterization and expansion

Bone marrow (BM) was collected from donors who underwent surgery at Rizzoli Orthopaedic Institute after obtaining informed consent, according to the protocol approved by the local Ethics Committee (approval Record No. 0004358 04/02/14).

BM harvesting was performed in the posterior iliac crest as previously described [32].Viable mononucleated cells (seeding density: 400000 cells/cm^2^) were seeded in complete medium composed of α-modified minimum essential medium (α-MEM; BioWhittaker, Lonza, Verviers, Belgium) supplemented with 20% lot-selected fetal bovine serum (FBS; Lonza, Basel, Switzerland), 1% GlutaMAX ^™^ (Gibco, Life Technologies, Paisley, UK). After 48 hr of culture, the medium was changed to remove non-adherent cells. When cells reached 70% - 80% confluence, they were detached by trypsinization (TripLe^™^ Select; Life Technologies) for 3 min at 37°C, counted and expanded at a seeding density of 4000 cells/cm^2^. Mesenchymal Stem/Stromal cells (MSCs) were maintained at 37°C in humidified atmosphere with 5% CO_2_, changing culture medium every 3 days. Cells were used for experiments until passage 6.

MSCs were tested for the Colony formation assay (CFU), the expression of CD14, 31, 34 and 45 that was < 3% and the expression of CD90 and CD73 that was > 95%. MSCs were induced *in vitro* to differentiate toward the osteogenic, adipogenic and chondrogenic phenotype. Osteogenic and chondrogenic differentiation was +++, while chondrogenic differentiation was ++. CFU, phenotype characterization and multilineage differentiation were performed as previously described in Pierini *et al*. [32].

### MSCs loading onto fibroin-alginate microcarriers

The day before cell loading, microcarriers were washed three times with saline solution, followed by two washes with culture medium, and finally suspended in culture medium to obtain a 50% (v/v) microcarrier suspension. Aliquots of microcarrier suspension were transferred in 2 ml sterile tubes and MSCs were added to the tubes and seeded onto fibroin-coated microcarriers at a density of 5000 cells/cm^2^ surface area [33]. The tubes were tightly closed and stirred on an oscillating shaker for 2 hr at 37°C-5% CO_2_ at 20 rpm to allow cell seeding. After the seeding, fresh medium was added in each tube. The following day, microcarriers were washed with saline solution to remove the unattached cells and moved on low attachment 24-well plate (Corning Costar, EuroClone). Seeded microcarriers were maintained in culture medium until day 8, changing the medium twice a week. The metabolic activity of adherent MSCs was performed by Alamar Blue Assay. In details, aliquots of cells seeded on the microcarriers were treated with 10% v/v Alamar Blue solution (Life Technologies) for 4 hr at 37°C. The fluorescence of the obtained solution was measured by Victor X3 (Ex/Em 560/590 nm).

### Staining procedures

To detect cellular motility of MSCs loaded into microcarriers, aliquots of microcarriers were incubated with of 2.5 μM green-fluorescent Calcein-AM (intracellular esterase detection, Thermo Fisher Scientific Inc., Whaltam, MA, USA) in saline solution for 10 min at 37°C and 5% CO_2_, washed several times with PBS to remove free Calcein-AM, and mounted as described in the next paragraph for LSFM observations and time-lapse imaging.

To test MSCs viability and distribution, the microcarriers were stained at the indicated time points with LIVE/DEAD^®^ (Thermo Fisher Scientific Inc., #L3224) Viability/Cytotoxicity Kit solutions (2.5 μM Calcein-AM and 10 μM Ethidium homodimer-1), according to the manufacturer’s protocol and by adding 5 μg/ml Hoechst33342 (Thermo Fisher Scientific Inc, #H3570, [1:2000]) to detect cell nuclei.

### Light sheet fluorescent microscopy (LSFM)

All the specimens were immersed in 1% low-melting temperature agarose gel (Sigma-Aldrich) warming at 37°C, and immediately loaded in a glass capillary (size 2 black, inner diameter of capillary ~1mm, #701932, BRAND GmbH). The capillary was continuously rotated manually for 5 min in order to guarantee the centrality of the specimen during the jellification phase. The glass capillary was mounted in the chamber, which was filled with complete medium without phenol red (DMEM + D-Glucose1g/L + pyruvate w/o L-Glutamine w/o phenol red, # 11880–028 Gibco). The samples were imaged using a Lightsheet Z.1 microscope (Carl Zeiss Microscopy GmbH, Jena, Germany), with 20×/1.0NA water-immersion detection optics and two-sided 10×/0.2 illumination optics, equipped with two PCO EDGE 4.2 cameras (sCMOS sensor, square pixels of 6.5×6.5 μm side length, 2048×2048 pixel resolution, 3-channel images, 16 bit dynamic range) (PCO AG, Kelheim, Germany). For Hoechst33342, Calcein-AM and Ethidium homodimer-1 imaging we used 1.5% (405 nm laser), 3.2% (488 nm laser) and 8.6% (561 nm laser) laser power and 50ms exposure time. The pivot scanner (Carl Zeiss) was used to deliver homogeneous illumination and, therefore, prevent shadows along the illumination axis. For all 3D datasets, a *z*-interval of 2 μm with a zoom of 0.40 was used. For time-lapse sequences, 200–500 *z*-slices were acquired for 10 hr, every 30 min, for a total amount of 21 *z*-stacks. The volumetric images were 1131×1131×1000 μm (1920×1920×500 pixels) in size, with 0.589×0.589×2.000 μm^3^ resolution. The chamber of the LSFM was maintained at 37°C, 95% relative humidity and 5% CO_2_ level during imaging. To counteract the degradation of the light sheet by the high amount of scattering, the specimen was sequentially illuminated through each of the two opposing illumination objectives, generating pairs of illuminated single-side images, and then combined into optical sections with a considerably improved penetration depth. All acquired LSFM raw data were processed using ZEN 2011 imaging software (Carl Zeiss, Germany). Optical sections were merged by maximum intensity projection for each *z*-stack and stacked groups of sections were merged to form a partial or a complete image of each sample. For full 3D reconstruction, the sample was then rotated by 90° and imaged again to obtain a sufficient resolution tangentially to the microsphere.The multi-view detection was combined into a single three-dimensional data set and reconstructed using ARIVIS software 2.10.4 (Carl Zeiss, Germany; Korea Basic Science Institute Chuncheon Center). Image acquisition and time-lapse were performed in triplicate.

### Tracking analyses

**Fig 1:**
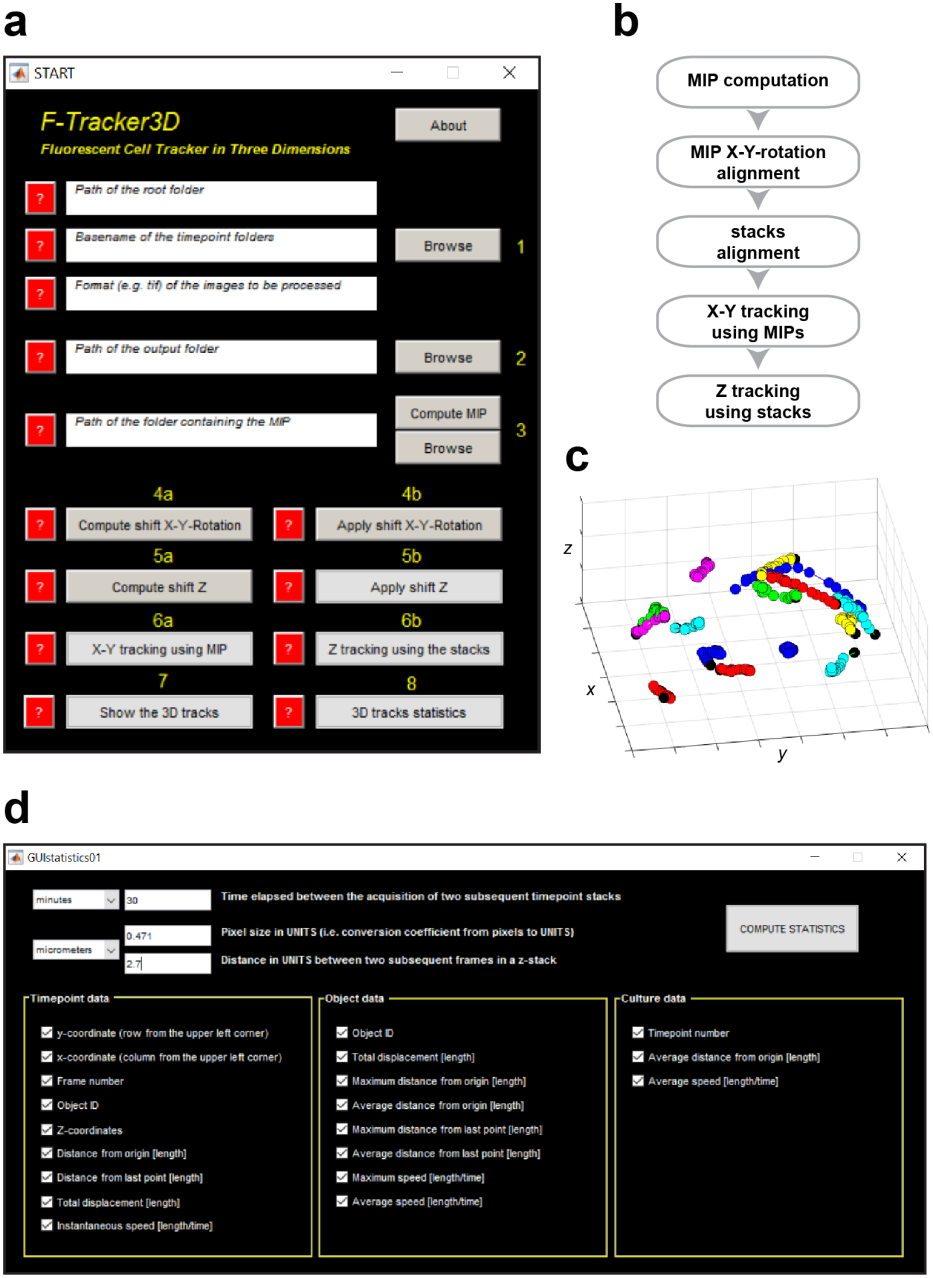
*F-Tracker3D*, an open source software tool for tracking cells in 3D. (**A**) *F-Tracker3D* GUI. (**B**) The *F-Tracker3D* flow chart: first of all, for each *z*-stack the MIP is computed along the *z*-dimension. The input stacks of fluorescence images are then rotated and aligned in (*x*, *y*, *z*) for setting a global absolute reference frame. MIPs are used to track the single cells in 2D. Finally, for each *z*-stack and (*x*, *y*) position, the *z* coordinate is estimated by selecting the *z*-plane containing the maximum intensity value. (**C**) Visualization of the 3D tracks. (**D**) A number of quantitative tracking measures can be automatically computed and exported as Microsoft Excel work sheet, MATLAB and csv files.

*F-Tracker3D* is written in MATLAB (The MathWorks, Inc., Massachusetts, USA). Source code and standalone executable version (*i*.*e*. not requiring MATLAB being installed) are freely distributed as an open-source software tool endowed with a Graphical User Interface (GUI, Fig 1A) at: https://sourceforge.net/p/f-tracker3d.

The S1 Video shows how to use *F-Tracker3D* to track cells moving into a spherical fibroin-alginate scaffold, and the tutorial together with the on-line content-oriented user manual makes using *F-Tracker3D* very natural. It may also be used for tracking tumour cells and many other different types of targets, similarly to what *CellTracker* does. Before tracking, the acquired images are corrected for uneven illumination, the so called “vignetting effect” [34], by using *CIDRE* [35]. Then, *F-Tracker3D* provides tools to rotate and align in (*x*, *y*, *z*) the input *z*-stacks of images for setting a global absolute reference frame (Fig 1B). For each *z*-stack, the 2D maximum intensity projection (MIP) is computed along the *z*-dimension and the single cells moving inside the spheroids are tracked in 2D using the MIP images and the methods provided by *CellTracker*. Alternatively, the full focused 2D images reconstructed from the *z*-stacks [36] can be used instead of MIPs. For each time instant *t* and (*x*, *y*) position, the *z* coordinate is finally estimated by analysing the whole stack of images and selecting the *z*-plane, which contains the maximum intensity value (Fig 1C). Path length, speed, distance from origin, and other quantitative tracking measures commonly found in the literature are automatically computed and exported to a Microsoft Excel work sheet (xls), MATLAB and csv files (Fig 1D).

## Results

### LSFM setup and cells adhesion observations

**Fig 2:**
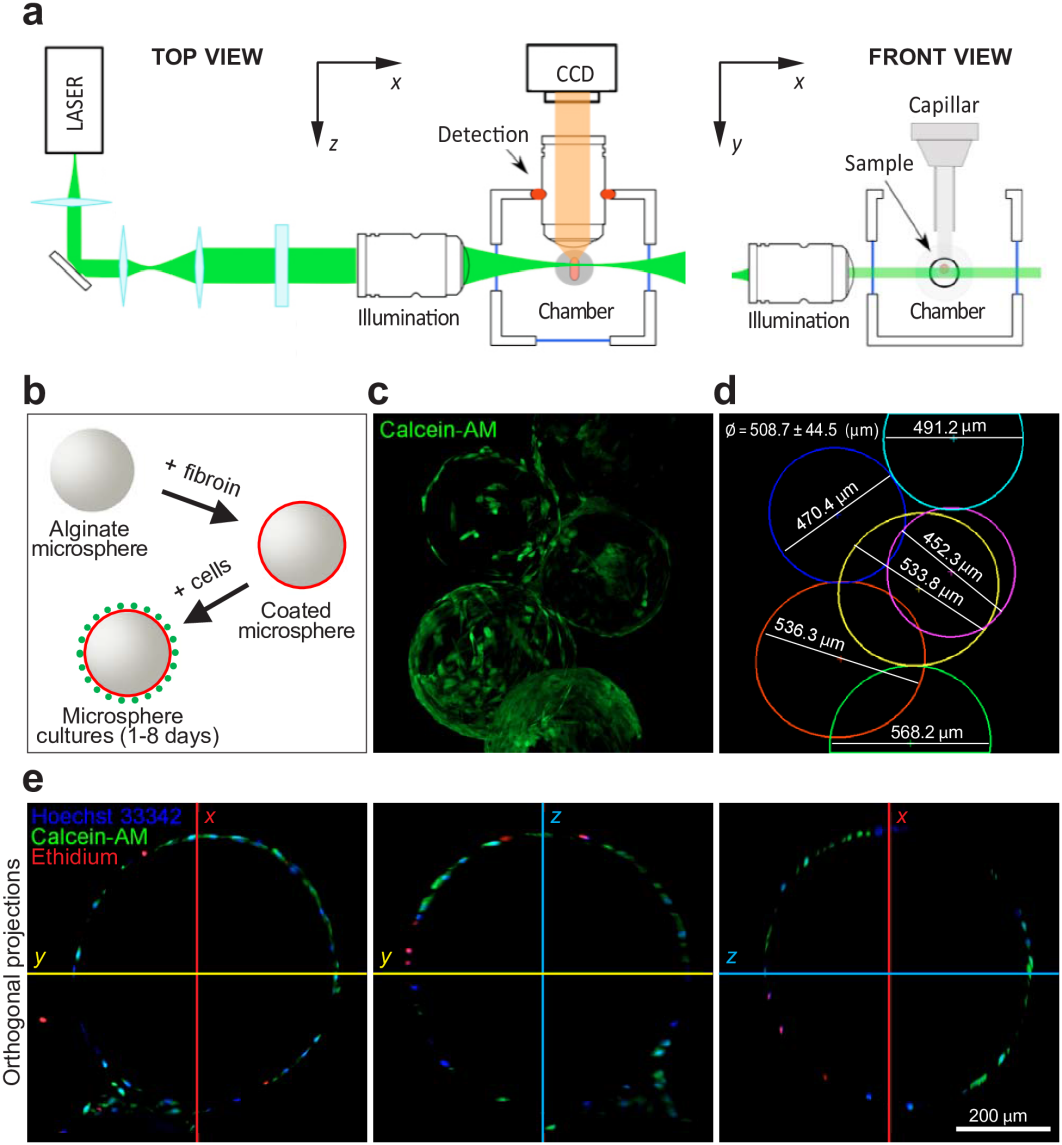
LSFM setup and characterization of MSCs loaded on fibroin-alginate microcarriers. (**A**) Schematic of the light sheet microscope detection objective lens, oriented at 90° with respect to the illumination direction. (**B**) Schematic representation of fibroin-alginate microcarriers preparation procedure. (**C**) Representative image showing several microcarriers loaded with MSCs stained with Calcein-AM to detect live cells (green channel). (**D**) Graphical view of microcarriers from **c** showing the size of the single spheres expressed as diameter in μm. (**E**) Orthogonal projections of a representative microcarrier showing the *x*, *y* and *z* single sections. Superimposed green, blue and red channels are shown for Calcein-AM to detect live cells (green channel), Ethidium homodimer-1 to detect dead cells (red channel) and Hoechst to detect cell nuclei (blue channel).

The settings we used for our LSFM imaging provided the excitation light laterally and the fluorophore emission light was collected through an objective positioned orthogonally to the illumination plane in order to reduce the background signal (Fig 2A).

To assess the feasibility of LSFM as an imaging tool for the analysis of cell adhesion capacity and viability on alginate scaffold coated with fibroin (Fig 2B), we first tested different mounting procedures onto capillaries MSCs-loaded microcarriers in agarose gel at different percentages, in order to find the best concentration to allow transparency, nutrient/gas exchange and the required stability of the specimen during motorized rotations or time-lapse imaging. According to these parameters, we selected the 1.5% agarose gel diluted in sterile water as previously described [37]. The microspheres maintained in a drop of buffer and mixed with the agarose into the cylindrical capillary (~1 mm of diameter) reach a final concentration of ~1% agarose once the sample is embedded. At lower agarose concentration (0.5% - 0.1%) an optical distortions and a typical drift of the agarose cylinder was observed during image acquisition.

To then perform LSFM imaging analyses, microcarriers loaded with MSCs were labelled with Calcein-AM, in order to visualize live cells. After 3 days of culture post initial loading, multiple microcarriers are clearly visible at 20× magnification, and it is possible to assess their sphericity and their aggregation probably due to the production of ECM proteins (Fig 2C). MSCs are able to adhere to the microcarrier surface although not homogeneously and variability is observed among the imaged microspheres. The FTIR spectrum confirmed the presence of silk fibroin in its stable conformation, due to the presence of typical absorption bands (at ~1620 cm^-1^ for Amide I and ~1520 cm^-1^ for Amide II) (Fig 2D). Interestingly, it is possible to observe multiple microspheres due to the formation of a network of intracellular connections that help the product aggregate (Fig 2C). The transparency of the alginate allows for the multi-sectioning of the specimen along the orthogonal axes (Fig 2E) at a higher resolution.

### Cell motility analysis

**Fig 3:**
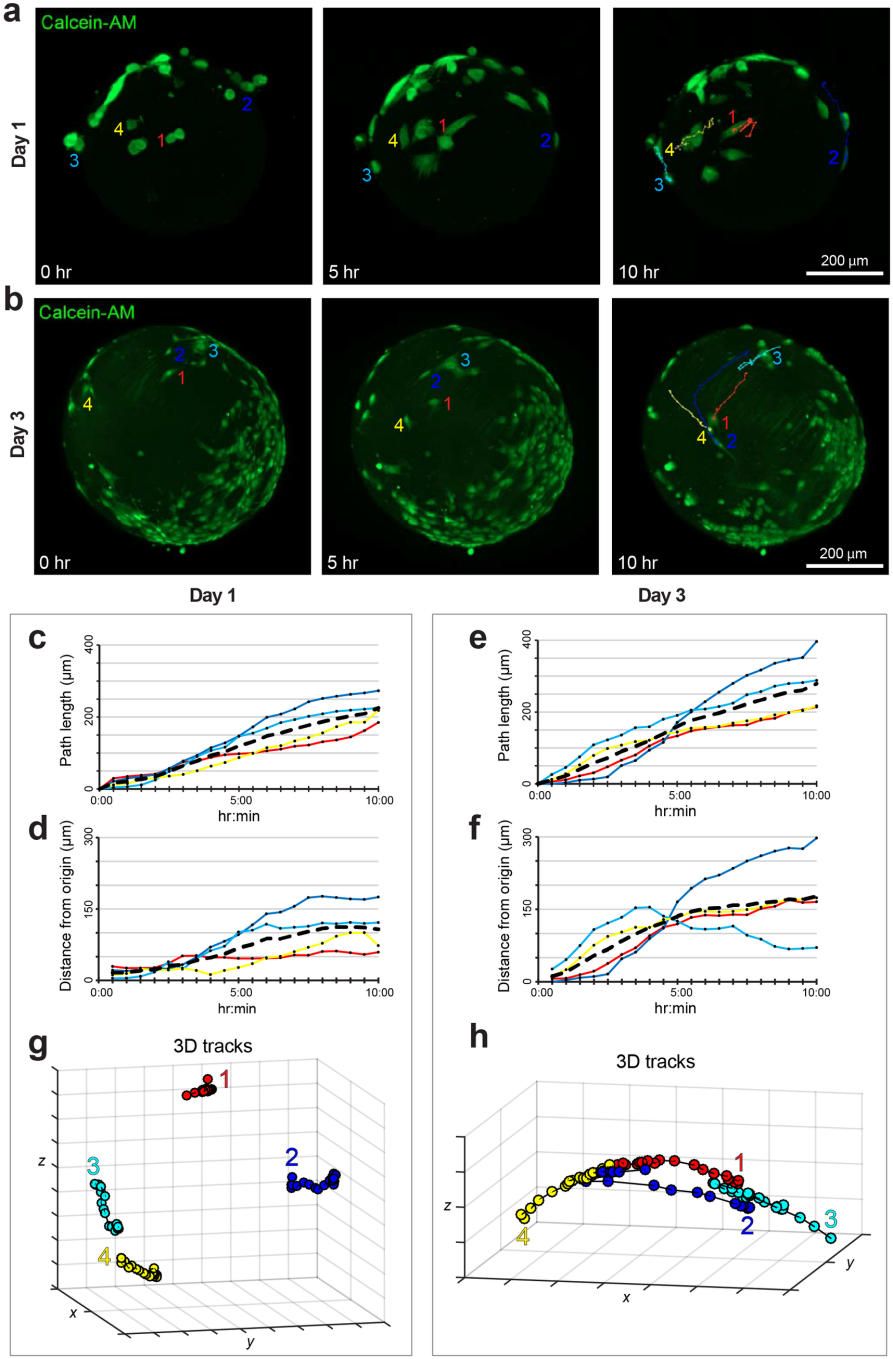
Cell motility analyses performed onto time-lapse LSFM imaging. (**A, B**) Representative frames from time-lapse S2 and S3 Videos for the evaluation of motility of MSCs loaded onto the microcarrier at day 1 and day 3 of culturing after the initial loading. Single green channel showing Calcein-AM positive cells is shown for 3 different time points (0 hr, 5 hr and 10 hr). (**C, D**) The graphs show the computed path length and distance from origin measured with *F-Tracker3D* of the cells marked in panel **A**. (**E, F**) The graphs show the computed path length and distance from origin measured with *F-Tracker3D* of the cells marked in panel **B**. The black dotted line represents the average value in each time point. (**G, H**) The graphs show the computed trajectories measured with *F-Tracker3D* of the 4 cells marked in panel **A** and **B**. Note: in all the graphs the curves referring to the corresponding single cells are reported in different colours.

In order to assess the cell motility, MSCs loaded microcarriers were stained and directly observed in time-lapse LSFM acquisitions during 10 hr, 1 day and 3 day intervals post initial seeding onto fibroin-alginate microcarriers (Fig 3, S2 and S3 Videos). We used *F-Tracker3D* to perform two different types of analyses. In the first analysis, for each time-lapse dataset we tracked the 4 most isolated cells, present in the central part of the sample and able to move without contact inhibition from surrounding cells. Then, we repeated the analysis by tracking for each time-lapse dataset a larger population of 15 cells, irrespective of surrounding cells.

One day after the initial seeding, the cells maintained a round shape and had not yet elongated (Fig 3A). By following the movements of the 4 most isolated cells (Fig 3A) the total length of the paths covered by cells was 200 μm on average (Fig 3B). However, it is interesting to notice that the cells didn’t follow a straight line moving away from the starting point, but rather turned over and returned towards the initial point, as depicted by the tracks (Fig 3G), by covering a distance from origin of about 100 μm (Fig 3D). After 24 h, the cells were, in fact, not fully attached and didn’t display the necessary polarization to cover a well-defined trajectory (S2 Video).

When the cell motility of 4 representative isolated cells was monitored 3 days after the initial seeding (Fig 3B), cells presented an elongated phenotype and thus the correct polarity to cover an oriented movement. The cells still covered a path length in the range of 250 μm (Fig 3E), but with an average calculated distance from the origin longer than the day 1 microcarrier of about 270 μm (Fig 3F), and they travelled along distinct linear trajectories (Fig 3H, S3 Video).

**Fig 4:**
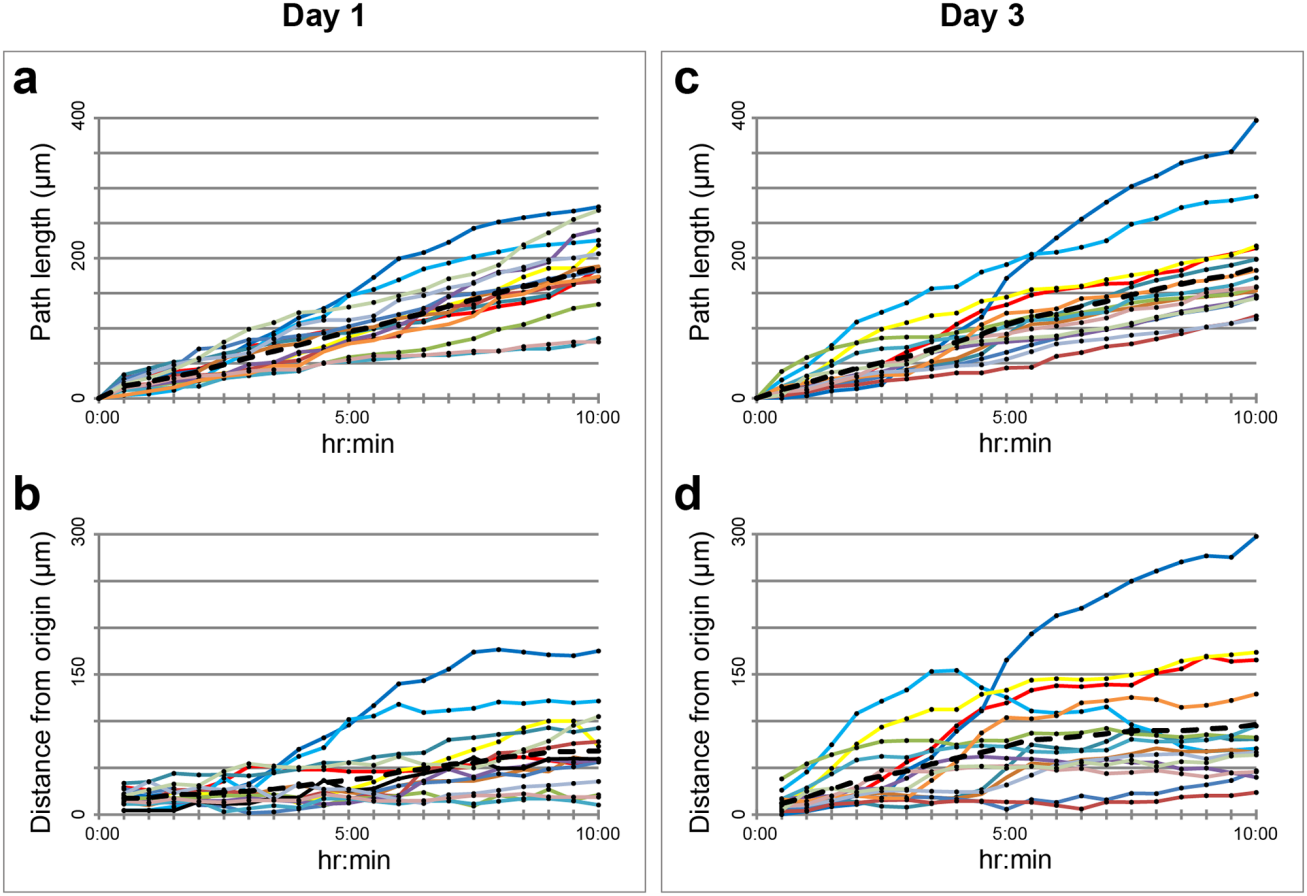
Large scale *F-Tracker3D* analysis on15 MSCs loaded onto fibroin-alginate microcarriers. The graphs show path length (**A, C**) and distance from the origin (**B**, **D**), computed considering populations of randomly selected cells (*n* = 15) at day 1 (**A, B**) and day 3 (**C**, **D**) from initial loading into microcarriers. Note: in the graphs the curves referring to the single cells are reported in different colours. The black dotted line represents the average value in each time point.

These results were confirmed when cells were chosen regardless of their confluence among each other. We thus randomly chose 15 cells from day 1 and day 3 MSCs-loaded microcarriers, and we tracked them for 10 hr (Fig 4). In both day 1 and day 3 post seeding, cells covered approximately 175 μm path length (Fig 4A and 4C), the average distance from the origin was approximately 50 μm for day 1 (Fig 4B), and 100 μm for day 3 (Fig 4D**)**, so considerably higher.

### Cell distribution analysis

**Fig 5:**
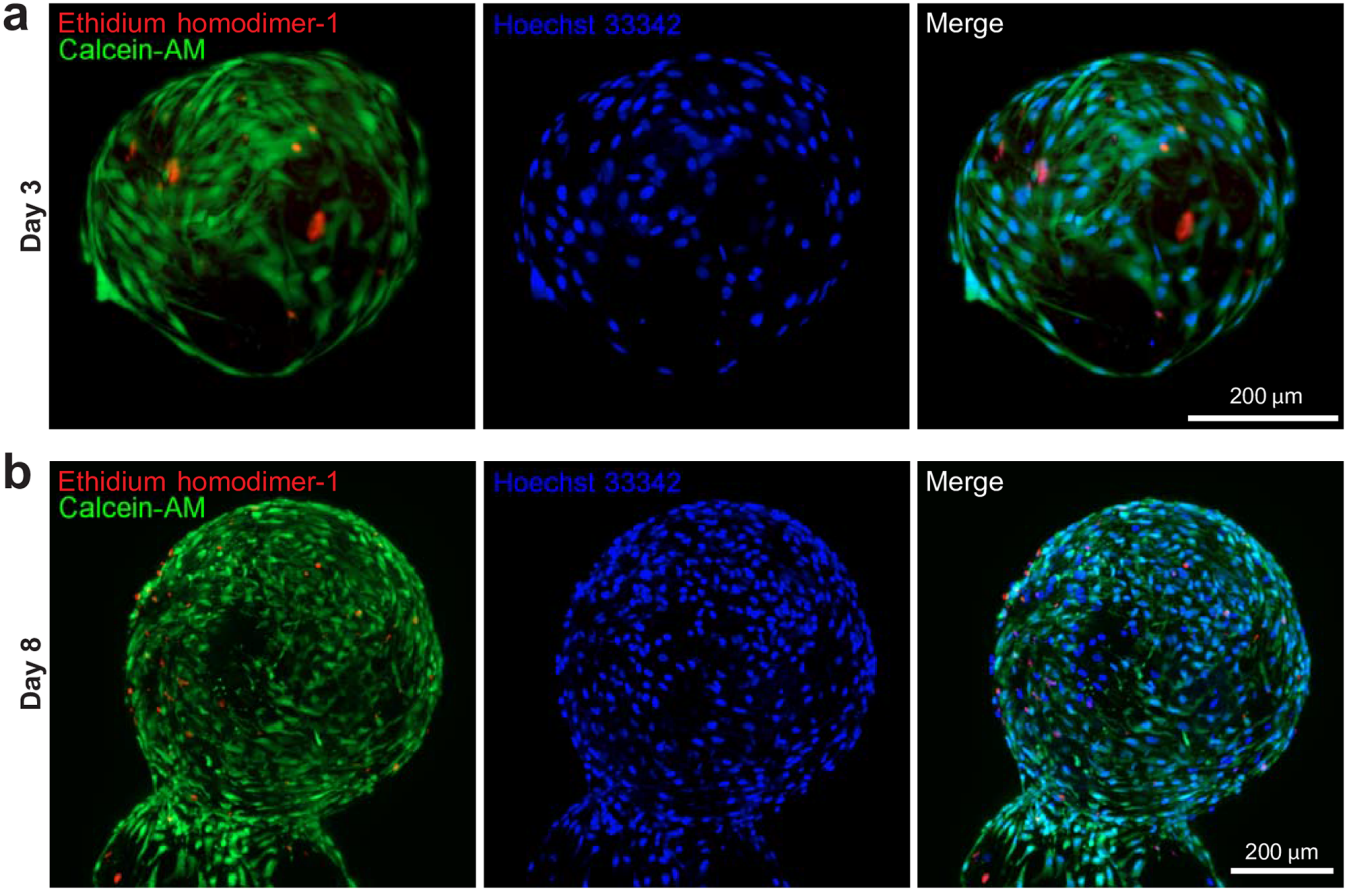
Cell distribution analysis performed onto single LSFM imaging. Representative images of MSCs loaded onto the fibroin-alginate microcarrier at day 3 (**A**) and 8 (**B**) of culturing after the initial loading. Superimposed green and red channels (Calcein-AM and Ethidium homodimer-1 respectively), single blue channel (Hoechst nuclear marker) and superimposed (merge) green, red and blue channels are shown.

To verify cell distribution over longer time periods in culture from the initial seeding, whole image acquisition with LSFM was performed at day 8 after MSCs loading onto fibroin-coated microcarriers and compared with day 3 (Fig 5). This analysis revealed that after 3 days MSCs start to cover the surface of the scaffold by elongating and stretching along the fibroin coating (Fig 5A), as also depicted by cell motility analysis (Fig 3B). As stained by Calcein-AM and Ethidium homodimer-1 markers, which allows one to distinguish live (green channel) and dead (red channel) cells, after few days of culturing most of cells are alive (Fig 5A). The low rate of cellular death is maintained post 8 days of culture, where the entire surface of the microcarrier is uniformly covered with living cells which did not penetrate the scaffold and remained on the surface of the spheroid (Fig 5B). Metabolic activity tested with Alamar blue showed a 200% increase of the optical density reading in comparison to values obtained one day after seeding, and this data is consistent with cell survival and proliferation.

Our setup enables the visualization of the 3D spherical architecture of the whole microcarrier and a detailed 3D volume reconstruction of the sample (S4 Video). The multiple views obtained along different angles can be combined into a single data set with an improved resolution by simply rotating the sample, so that hidden parts of the sample become visible.

## Discussion

Imaging techniques for 3D analysis have been identified as a strategic priority in tissue engineering and regenerative medicine research [38]. In our study, we propose LSFM technique as an innovative tool to overcome the limitations of the current imaging techniques. As far as we know, we are the first to use LSFM to characterize MSCs adhesion, distribution and motility on a microcarrier and to combine LSFM and a 3D tracking system in order to investigate the behavior of MSCs once seeded onto those scaffolds.

The proof of principle was performed onto silk-fibroin based alginate microcarriers, to verify the acquisition of cells adhesion capacity of alginate after a protein based surface modification is made with fibroin.

Our LSFM set up allowed us to track the same cells over the spheres for a long period of time, maintaining cell viability without photo-damage or photo-bleaching from the fluorophores derived from the Calcein-AM, Ethiudim homodimer-1 and Hoechst staining. In addition, the ability to simultaneously visualize different markers using multicolour staining approaches is essential when characterizing different statuses of cells in culture. The acquired 3D images clearly identify the whole microcarrier structure, enabling one to determine the cell distribution on the entire microscaffold. In addition, we could observe the motility of single cells and perform computational measurements and produce 3D movies.

Only a few tools are currently available for tracking cells in 3D using *z*-stack of fluorescent images [26]. The most noteworthy solution is the *ImarisTrack* module of *IMARIS*. *ImarisTrack* offers a wide choice of multiple automatic tracking algorithms and provides researchers with a set of tools to support their analyses. Cells/particles are tracked in two steps: first, the objects of interest are detected using one or more of the available wizard-driven segmentation tools, then, objects at consecutive time points are related and integrated into a single moving object. Aside from the manual tracking option, three different algorithms have been implemented for this purpose. Although *ImarisTrack* represents the most complete suite currently available, advanced image processing knowledge is needed to correctly execute the single stages and appropriately combine them for an effective analysis of the input datasets. Furthermore, *IMARIS* is a commercial software that is not available to all researchers. The most common public solution for tracking cells/particles is *ImageJ* [39]. In *ImageJ*, a few plug-ins are available for this purpose. Among them, *MTrackJ* (www.imagescience.org/meijering/software), *ParticleTracker* [40], and *TrackMate* [41] are noteworthy. *MTrackJ* is designed for manual cell tracking. The user must define the (*x*, *y*, *z*) positions for each *t*, resulting in an extremely time consuming procedure. On the other hand, *ParticleTracker* also provides some automatic methods for tracking brightness particles. A recent comparative study has evaluated *ParticleTracker* as one of the best particle tracking options [42]. However, it was designed to track pre-segmented particles, and its usage with cells in 3D applications, such as cells moving on a 3D scaffold, yields particularly complex results. *TrackMate* is the freely available solution that better resembles the segmentation and tracking wizard of *IMARIS*. It is designed to track spots and roughly spherical objects following a two-step scheme where the segmentation and particle-linkage steps are separated. Each step is managed in the user interface by a specific panel, and basic programming skills are required for usage. However, *TrackMate* does not provide any solution for aligning different *z*-stacks of images, therefore requiring other plug-ins or external programs.

In order to provide biologists with a turnkey solution to monitor cell motility on MSCs-loaded microcarriers, we designed *F-Tracker3D*, an open-source user-friendly software tool for automatically tracking individual fluorescent tagged proteins, cellular structures, organelles and cells in 3D without requiring the user to perform any segmentation stage. Accordingly, no image processing skills or knowledge of the sample is needed. The program simply requires as the input a set of *z*-stacks of fluorescence images acquired in time-lapse, by confocal microscopy or LSFM. For each tracked cell it automatically provides (*x*, *y*, *z*) for each time point *t*, and several measurements computed according to the spatial cell displacement.

By combining LSFM and *F-Tracker3D*, we were able to demonstrate that the motility of MSCs one day after the seeding onto fibroin-coated alginate scaffolds is different compared to 3 days post seeding. One day after seeding on fibroin coated alginate beads, the morphology of the cells are round, typically of cells not yet fully adherent to the surface material. After 3 days, cells start to produce ECM proteins that allows full attachment to the fibroin coating and also provides the adhesive strength that determines the polarized orientation for motility along the surface of the microcarriers [43][44].

This data could be helpful and suggest that the analyses on cell-biomaterial interactions should be performed several days after cells seeding to allow perfect cell adhesion and elongation along the surface of the desired biomaterial. There may be limitations if cells are seeded on a different type of scaffold. In particular, one of the major limits of LSFM is the light penetration into thick and scattering samples. If parts of the samples have a significantly higher refractive index (*e*.*g*. lipid vesicles, membranes, organelles), they can also lead to a focusing effect resulting in degradation of image quality. To overcome this problem, the light sheet can use dual side illumination and the pivot scan that reduces the presence of shadows behind the scattering structures, or can be combined with two-photon excitation, which improves the accessible imaging depth to hundreds of micrometers and reduces sample-induced aberrations [45]. An alternative approach is the optical clearing, a procedure that renders the tissue transparent to light by removing the main scattering source. Although these methods guarantee high transparency, their applicability is limited by protein fluorescence quenching, tissue shrinkage, and incompatibility with live imaging [46].

In our study, we present microcarriers with a high degree of transparency that minimize the refractive index mismatch between medium and cellular organelles and uniformly maintain the intensity distribution along the light sheet excited signal without chemical clearing methods.

## Conclusions

The method we propose is holistic, non-invasive, non-destructive, and quantitative. It enables a 3D analysis of the entire cell-loaded scaffold. With the combination of LSFM and *F-Tracker3D*, we were able to detect the adhesion capacity of a modified alginate-based scaffold material and the differences in cell motility along the microcarrier surfaces. Moreover, we were also able to produce a holistic view of the cell distribution during time. We therefore propose this imaging technique as a tool for the design of tissue engineering products, as well as for quality control during production process validation, from the fabrication to the functionalization of a scaffold to be used as a medical device.

## Supporting information

S1 Video F-Tracker3D video tutorial. A video tutorial showing how to use *F-Tracker3D* to easily track cells moving into a 3D spherical object. A sample dataset to test the software is provided at: https://sourceforge.net/p/f-tracker3d.
(AVI)

S2 Video Time-lapse LSFM imaging of MSCs at day 1. Movie of Calcein-AM stained cells (green channel) at day 1 from initial seeding, imaged during 10 hr time-lapse acquisition (21 *z*-stacks of images, a *z*-stack acquired every 30 min).
(AVI)

S3 Video Time-lapse LSFM imaging of MSCs at day 3. Movie of Calcein-AM stained cells (green channel) at day 3 from initial seeding, imaged during 10 hr time-lapse acquisition (21 *z*-stacks of images, a *z*-stack acquired every 30 min).
(AVI)

S4 Video 3D rendering LSFM imaging of MSCs. Representative 3D rendering movie of a microcarrier loaded with MSCs (Calcein-AM positive cells green channel, Ethidium homodimer-1 red channel, and Hoechst blue channel) after days 8 of culture from the initial loading.
(AVI)
